# The Association between Primary Open-Angle Glaucoma and Blood Pressure: Two Aspects of Hypertension and Hypotension

**DOI:** 10.1155/2015/827516

**Published:** 2015-10-18

**Authors:** Hye Jin Chung, Hyung Bin Hwang, Na Young Lee

**Affiliations:** ^1^HanGil Eye Hospital, Incheon 21388, Republic of Korea; ^2^Department of Ophthalmology, Incheon St. Mary's Hospital, College of Medicine, The Catholic University of Korea, Seoul 403-720, Republic of Korea

## Abstract

Glaucoma is the second leading cause of blindness worldwide. Although the mechanism of the development of primary open-angle glaucoma (POAG) is not fully understood, elevated intraocular pressure (IOP) is considered the most important risk factor. Several vascular factors have also been identified as risk factors and can lead to hypoperfusion of the optic nerve head and thus may play an important role in the pathogenesis and progression of POAG. The results of the present study suggest that both high and low blood pressure (BP) are associated with an increased risk of POAG based on a comprehensive literature review. Elevated BP is associated with elevated IOP, leading to increased risk of glaucoma, but excessive BP lowering in glaucoma patients may cause a drop in ocular perfusion pressure (OPP) and subsequent ischemic injury. The relationship between IOP, OPP, and BP suggests that the relationship between BP and glaucoma progression is U-shaped.

## 1. Introduction

Glaucoma is commonly defined as optic neuropathy characterized by progressive loss of retinal ganglion cells (RGCs) which is associated with characteristic structural damage to the optic nerve and visual field loss. Risk factors related to glaucoma include intraocular pressure (IOP), age, family history, clinical appearance of the optic nerve, race, and potential vascular disease [1–4].

Although the mechanism of RGC death is not fully understood, elevated IOP is considered the most important risk factor [5, 6]. Several large randomized clinical trials showed a relationship between IOP and glaucoma development and progression [5–9]. Besides the mechanical effect of raised IOP on the optic nerve head (ONH), several vascular factors have also been identified as risk factors [10]. Such factors can lead to hypoperfusion of the ONH and may thus play an important role in the pathogenesis and progression of primary open-angle glaucoma (POAG) [11–15].

Among vascular factors, systemic hypertension may contribute to increases in IOP via overproduction or impaired outflow of aqueous humor [16]. However, the relationship between glaucoma and blood pressure (BP) remains under debate. While some studies report that systemic hypertension is a risk factor for glaucoma [4, 17, 18], other studies indicate that low systemic BP is a risk factor for the development and progression of glaucoma. A direct and clear relationship between glaucomatous damage and BP level has not been established [19]. Moreover, the association between BP and IOP is inconsistent.

Some but not all population studies found statistically significant positive associations between systolic blood pressure (SBP) and diastolic blood pressure (DBP) with IOP [3, 4, 20–28].

In the present study, we reviewed the relationship between POAG and BP, focusing on two aspects: hypertension and hypotension.

## 2. Method of Literature Search

The Medline database was used for the literature search in this review. Although every effort was made to use the most recent references possible, articles were used irrespective of the year of publication if deemed appropriate. The key words searched included the following: intraocular pressure, ocular perfusion pressure, glaucoma, blood pressure, circadian fluctuation, and risk factors. After retrieving relevant articles using these key words, a search was conducted through the studies cited in these articles, and additional papers were identified. Abstracts of papers in languages other than English were also surveyed. Medical Subject Headings (MeSH) searches were also performed. Case reports and abstracts from meeting presentations were excluded.

## 3. Blood Supply of ONH

The ophthalmic artery, which is the first branch of the internal carotid artery, gives off 2–4 posterior ciliary arteries. Posterior ciliary arteries later divide into 10–20 short posterior ciliary arteries that pierce the sclera and enter the globe around the optic nerve. It was reported that the superficial layers of the ONH are supplied by the central retinal artery while the deeper prelaminar regions are supplied by the posterior ciliary arteries, which branch off the circle of Zinn-Haller [16].

ONH circulation is thought to be anatomically and physiologically similar to the circulation in the retina, which is characterized by tight junctions, abundant pericytes, and nonfenestrated endothelium [29]. The capillaries of ONH do not leak fluorescein and may represent a nerve-blood barrier, supporting the concept of the retina-nerve vasculature as a continuous system with the central nerve system [29, 30]. Histologic examination of glaucomatous optic nerves showed a reduction in the number of capillaries, consistent with the degree of neural loss.

Blood flow in the anterior optic nerve depends on many factors, which include the ocular perfusion pressure (OPP) and the resistance to flow as determined by the vascular caliber in the arterioles and capillaries [31]. The ability to keep local tissue blood flow constant and counteract changes in the local metabolic environment is called autoregulation [32]. Moderate increments in IOP and systemic BP have little effect on anterior optic nerve-blood flow, and autoregulatory mechanisms maintain flow in hyperoxic and hypercapnic conditions. In contrast to the extraocular and choroidal vessels, retinal vessels have no neural innervation. Therefore, local vascular mechanisms are mainly responsible for matching perfusion to the changes in metabolic demand [33, 34]. The process of autoregulation in a vascular bed maintains constant or nearly constant blood flow through a wide range of perfusion pressures. However, if autoregulation is impaired, elevated IOP may reduce optic nerve perfusion. The circulatory networks of the optic nerve and retina have deficient autoregulation in POAG.

Substances produced by the vascular endothelium play a major role in the control of ocular blood flow, and these include the vasodilators nitric oxide and prostacyclin and vasoconstrictors such as angiotensin and the endothelins [35].

Regulation of blood flow through the choroid is under the control of the autonomic nervous system. Data regarding choroidal autoregulation are contradictory. The autonomic tonus may protect the eye from transient elevations in systemic BP under normal circumstances; however, the autonomic nervous regulation may break down in the presence of systemic hypertension.

## 4. BP and IOP

**Figure 1:**
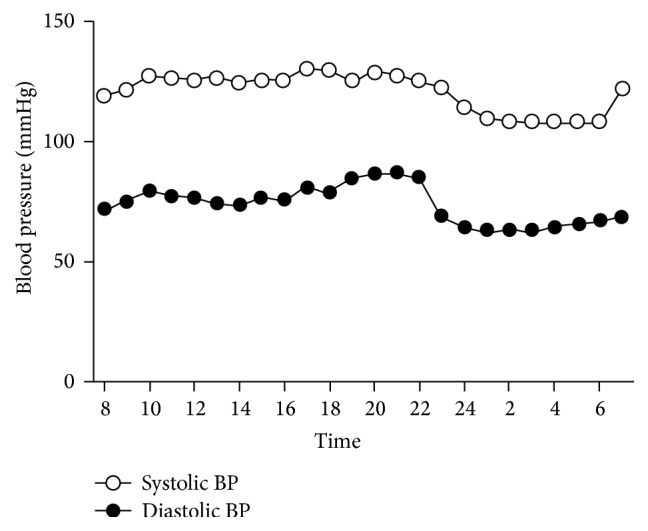
Circadian curve of mean systolic and diastolic blood pressure (BP) in 34 combined dipper and nondipper patients. Modified from Quaranta et al. [36] and Staessen et al. [39].

BP is one of numerous metabolic systems in humans that exhibit a circadian rhythm [36–39] (Figure 1). Millar-Craig et al. showed that BP is lowest at around 3 AM and increases gradually during the early morning hours before waking, reaching a peak at midmorning [36, 37]. These fluctuations have been attributed to the nocturnal decrease in sympathetic activity and circulating catecholamine levels. In humans, resting levels of plasma epinephrine and norepinephrine (markers of sympathetic nervous activity) exhibit endogenous circadian rhythmicity with a broad peak during the middle of the biological day, and the BP rise that begins before waking is independent of behaviors [40].

**Figure 2:**
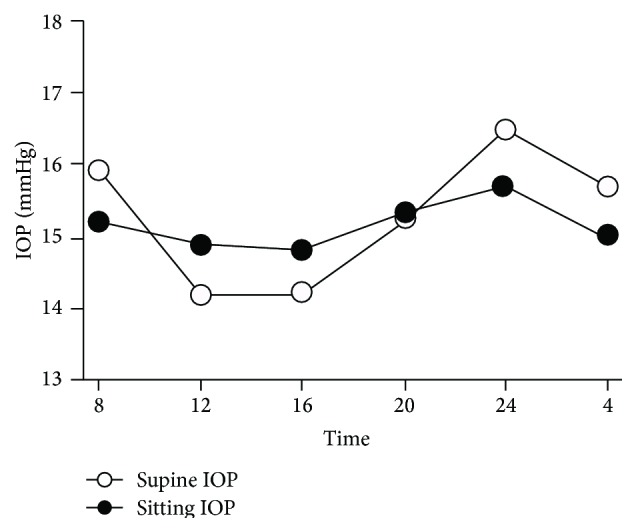
Circadian curve of mean supine and sitting intraocular pressure (IOP). Modified from Fogagnolo et al. [41].

Circadian variations in IOP also exist, and many studies were conducted to characterize these rhythmic patterns. The traditional view is that IOP is generally higher in the morning, but recent research in both healthy and glaucomatous eyes questioned this pattern [36, 40, 41] (Figure 2).

Lui et al. performed IOP measurements every 2 hours over 24 hours in young healthy volunteers [40]. The average IOP was significantly higher in the dark period than in the light-wake period. In comparison with the sitting IOP values in the first group, the supine IOP in the second group was significantly higher during the light-wake period. The authors concluded that a nocturnal IOP elevation can appear independent of body position change, but change in posture from upright to recumbent may contribute to the relative nocturnal IOP elevation. Another study showed that there were no significant changes in supine IOP at any time point, although the IOP peaked at midnight (16.5 mmHg) and troughed at noon and 4 PM (14.2 mmHg), nor any significant changes in sitting IOP over time (mean values between 14.8 mmHg and 15.7 mmHg). The same was true when the daytime and nighttime measurements were compared [41]. In addition, a recent study confirmed that 24-hour IOP fluctuations were not highly reproducible and that IOP patterns were not sustained from day to day in healthy young volunteers [42]. Unlike circadian rhythm of BP, controversy exists on the circadian IOP cycle.

The majority of population-based studies reported a positive association or correlation between SBP, DBP, and IOP [20, 21, 26–28, 43–45]. A recent meta-analysis showed a pooled average IOP increase of 0.26 mmHg (95% CI, 0.23–0.28; *I* ^2^, 42.5%) and 0.17 mm Hg (95% CI, 0.11–0.23; *I* ^2^, 91.2%) associated with a 10 mmHg and 5 mmHg increase in DBP, respectively, with similar results seen in cross-sectional and longitudinal studies [46]. These trends may be because systemic hypertension increases IOP via overproduction or impaired outflow of aqueous humor [16].

Several studies investigated the vascular risk factors in the pathogenesis of glaucoma, with BP and OPP being the most studied. The vascular hypothesis is based on the assumption that abnormal perfusion and the subsequent ischemia of the ONH play a major role in the loss of RGCs.

OPP can be defined as the systolic, diastolic, or mean OPP. The mean OPP (MOPP) can be calculated as 2/3 of the mean arterial BP-IOP, where mean arterial pressure = DBP + 1/3(SBP − DBP). The factor of 2/3 accounts for the drop in BP between the brachial and ophthalmic artery when the subject is seated [47]. Systolic OPP is defined as the difference between the systemic SBP and IOP, whereas diastolic OPP (DOPP) equals the systemic DBP-IOP [48]. DOPP is especially useful for displaying the lowest OPP values and is regarded as an independent risk factor for open-angle glaucoma (OAG).

When calculated by this equation, a certain change in IOP or BP results in the same value of MOPP. However, an experimental study showed that IOP is more important than BP in determining retinal function and that, for a given OPP, a higher IOP elevation induces greater retinal dysfunction [49]. This is possibly because BP modification influences vascular supply only, whereas an IOP elevation affects the vascular supply via a reduction in OPP and produces mechanical stress on retinal neurons which is OPP independent.

## 5. POAG and Hypertension

As previously mentioned, data on the association between hypertension and IOP is consistent across studies. However, the relationship between POAG and BP is complex and poorly understood. Several large-scale epidemiologic studies investigated this relationship, with most studies describing conflicting reports. Several studies reported a low risk of glaucoma in individuals with elevated BP [50–53], whereas others reported significant associations between high systemic BP and POAG using cross-sectional data [17, 44, 45, 54]. However, the Barbados Eye and the Proyecto VER studies failed to demonstrate a significant relationship between BP and POAG [55, 56].

Although the influence of systemic hypertension on glaucoma is complex, several mechanisms are suggested. The Baltimore Eye Survey showed an age-related association between BP and glaucoma [2]. In younger patients, hypertension showed a protective effect that might improve OPP. However, in older patients, this positive effect is lost and an increased risk of glaucoma is seen, most likely as a result of blood vessel alterations induced by arterial hypertension with disturbed oxygen and nutrition supply [57]. In systemic hypertension, chronically elevated BP may result in arteriosclerosis, changes in the size of the precapillary arterioles, and capillary dropout leading to increased resistance to blood flow and, thus, reduced perfusion [58]. Also, disruption of the autoregulatory mechanisms of blood flow in the ONH vascular beds at high levels of BP may further contribute to reduced perfusion, which may counteract any protective effect afforded by higher perfusion pressure [13]. These findings lead to the assumption of a U-shaped relationship between BP and the progression of glaucoma [54].

Another important consideration is the relationship between BP, IOP, and POAG. Elevated IOP is considered the most important risk factor for the development and progression of POAG. Therefore, the relationship between BP and IOP should be considered when evaluating the association between POAG and hypertension. Moreover, OPP is regarded as another important risk factor for disease development and progression. As previously mentioned, as OPP includes IOP, it is possible that some of the findings attributed to OPP are in fact exclusively secondary to IOP. Therefore, it is always important to verify whether previous studies adjusted for IOP. Several large epidemiology studies that adjusted for IOP are shown in Table 1. Interestingly, Memarzadeh et al. showed no association between OAG and conventionally defined systemic hypertension; however, the relationship was found across a range of BPs rather than by arbitrary divisions and definitions. Elevated systolic and mean arterial BPs were significantly associated with a high prevalence of OAG, independent of the impact of IOP.

## 6. POAG and Hypotension

Pache and Flammer reported hypotension, and in particular a nocturnal drop in BP, as an important risk factor for OAG [59]. Randomized clinical trials also suggested that low BP is associated with risk and progression of glaucoma. In the Early Manifest Glaucoma Trial, lower SBP in patients with lower baseline IOP was associated with faster progression to OAG [50]. However, this J-shape association between systolic and diastolic BP and IOP may be confounded by antihypertensive treatment status, as treated or overtreated hypertensive patients can have a normal or low BP but elevated POAG risk [46]. In the Thessaloniki Eye Study, low DOPP was associated with an increased risk for POAG in subjects undergoing antihypertensive treatment [51]. In the Baltimore Eye Study, a DOPP of less than 35 mmHg was associated with a significant increase in the prevalence of glaucoma [2]. In the Egna-Neumarkt Study, the prevalence of glaucoma decreased progressively with increased DOPP, whereas no correlation was detected with either systolic or mean OPP [17].

In terms of the association between BP and glaucoma, nocturnal hypotension may exacerbate the progression of visual field loss in patients with glaucoma [60, 61]. When a nocturnal BP dip coincides with an IOP spike, a substantial OPP reduction is thought to produce an intermittent insult that increases the risk of disease progression [62]. DOPP is especially useful for displaying the lowest OPP values and is regarded as an independent risk factor for OAG. A recent study suggested that nocturnal BP could be a modifiable risk factor for glaucoma severity and progression [63]. Nocturnal hypotension is caused primarily by sleep, presumably owing to sympathetic withdrawal. However, physiologic nocturnal hypotension is regarded as a protective mechanism during sleep; therefore, artificial regulation of nighttime BP should be considered with caution.

## 7. Conclusion

Several studies demonstrated that both high and low BP are associated with increased risk of POAG. An increase in BP is associated with an elevated IOP, leading to increased risk of glaucoma. In addition, the microangiopathy of hypertension can result in end organ damage including the retina and optic nerve. Hypertension must be treated because it is one of the most important risk factors for cardiovascular morbidity and mortality. But excessive BP lowering in glaucoma patients may cause a drop in OPP and subsequent ischemic injury. In particular, DOPP is useful for displaying the lowest OPP values and is regarded as an independent risk factor for OAG. Although low OPP is an established risk factor in POAG, as OPP includes IOP, it is possible that some of the findings attributed to OPP are in fact exclusively secondary to IOP. Current treatment of POAG aims to reduce IOP; however, there is no evidence to support the value of increasing BP as therapy for POAG. Such recommendations are not currently warranted, since we lack crucial information about the microvascular beds in which perfusion is important in glaucoma, and the appropriate methods to evaluate their blood flow [16]. More research on treatments designed to increase OPP by increasing BP is needed.

The relationship between IOP, OPP, and BP may be related to a U-shaped relationship between BP and the progression of glaucoma. Therefore, both high and low BP should be monitored with caution especially in patients with progressive glaucoma despite controlled IOP.

## Figures and Tables

**Table 1:** Characteristics of the studies investigating the association between primary open-angle glaucoma (POAG) and blood pressure (BP) with adjustment for intraocular pressure (IOP).

Reference	Country	Sample size	Study design	Exposure	Outcome
Blue mountains eye study [44]	Australia	3654	Population-based survey	HTN	OAG
The Beijing eye study [20]	China	3222	Population-based survey	HTN	POAG
The Singapore Malay eye study [64]	Singapore	3280	Cross-sectional population-based study	SBP, DBP, HTN	POAG
Los Angeles Latino eye study [54]	USA	6130	Cross-sectional population-based study	SBP, DBP	OAG
Barbados eye study [52]	India	3222	Cohort study of prospective population-based study	SBP, DBP, HTN	OAG
The Rotterdam study [65]	Netherlands	5317	Cross-sectional prospective population-based study	SBP, DBP	OAG

HTN, hypertension; SBP, systolic blood pressure; DBP, diastolic blood pressure; OAG, open-angle glaucoma; POAG, primary open-angle glaucoma.
